# Advances in Metabolic Engineering of Cyanobacteria for Photosynthetic Biochemical Production

**DOI:** 10.3390/metabo5040636

**Published:** 2015-10-27

**Authors:** Martin C. Lai, Ethan I. Lan

**Affiliations:** 1 Undergraduate Honors Program of Nano Science and Engineering, National Chiao Tung University, Hsinchu 300, Taiwan; E-Mail: martinnctu@hotmail.com; 2 Department of Biological Science and Technology, National Chiao Tung University, Hsinchu 300, Taiwan

**Keywords:** metabolic engineering, cyanobacteria

## Abstract

Engineering cyanobacteria into photosynthetic microbial cell factories for the production of biochemicals and biofuels is a promising approach toward sustainability. Cyanobacteria naturally grow on light and carbon dioxide, bypassing the need of fermentable plant biomass and arable land. By tapping into the central metabolism and rerouting carbon flux towards desirable compound production, cyanobacteria are engineered to directly convert CO_2_ into various chemicals. This review discusses the diversity of bioproducts synthesized by engineered cyanobacteria, the metabolic pathways used, and the current engineering strategies used for increasing their titers.

## 1. Introduction

**Figure 1:**
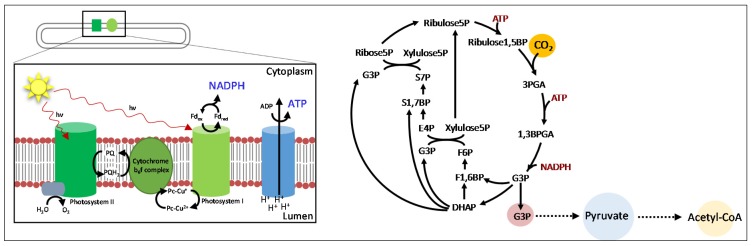
Schematics of cyanobacterial central metabolism. NADPH and ATP are generated through light reactions. Subsequently, they are used to convert CO_2_ into central metabolites, which can be used for biochemical synthesis.

Increasing concerns over energy and environmental problems prompted the need to develop renewable chemicals and fuels. Advances in genetic manipulation and genomics understanding enabled rapid advancement of microbial cell factory development. Once the enzymatic and genetic information for metabolic pathways producing important commodity biochemicals are solved, these genetic parts are then transferred to other organisms capable of utilizing diverse bioresources for growth. Among the various microbial systems, cyanobacteria have received an enormous amount of attention in recent years. Cyanobacteria are photosynthetic prokaryotes that grow through the direct utilization of sunlight and CO_2_. Cyanobacteria generate ATP and NADPH through oxygenic photosynthesis, and subsequently use them to fix CO_2_ into central metabolite through the Calvin–Benson–Bassham (CBB) cycle (Figure 1). By tapping into the metabolism of cyanobacteria, central metabolites may be directly transformed into desired biochemicals. This approach bypasses the need of repeated construction and deconstruction of plant biomass, which potentially increases the overall solar energy conversion efficiency.

Most commonly used cyanobacterial models (Table 1) are *Synechococcus elongatus* PCC 7942, *Synechocystis* PCC 6803, *Synechococcus* sp*.* PCC 7002, and *Anabaena* sp. PCC 7120 (hereafter referred to as PCC 7942, PCC 6803, PCC 7002, and PCC 7120, respectively). The genomes of these model cyanobacteria have been sequenced. In addition, these sequences have been organized and compiled into databases, such as cyanobase [1], for rapid access. The construction of these genome databases enabled the interpretation of cyanobacterial metabolism and facilitated the development of recombinant engineering. In addition, metabolomics analyses on these cyanobacteria have been reported [2,3,4]. This information, together with the construction of metabolic models [5,6,7], is useful for both understanding the basic metabolism of cyanobacteria and achieving higher level of metabolic redirection and control. PCC 7942 and PCC 6803 are freshwater cyanobacteria, while PCC 7002 is a marine species with the additional advantage of having higher salt tolerance. PCC 7120 is diazotrophic, which means it can utilize N_2_ as nitrogen source. With the exception of PCC 7120, all other model strains are naturally competent [8,9]. PCC 7120 transformation have been carried out through conjugation [10].

Cyanobacterial carbon and energy metabolisms differ significantly from that of heterotrophs commonly used in fermentation. As a result, additional metabolic engineering strategies such as changing reducing cofactor preference from NADH to NADPH, expressing pathways with higher thermodynamic driving force, and expressing transporters for excretion of products are often needed. Here we summarize the advances made in the metabolic engineering of cyanobacteria for the production of biochemicals and discuss the strategies and pathways used to improve their productivities. Table 2 is a comprehensive list containing biochemical targets, genes and promoters used, and production titers. The relevant metabolites associated with each biochemical target are also included in Table 2.

**Table 1:** Model cyanobacteria strains.

Strain	*Synechocystis* PCC 6803	*Synechococcus* sp.PCC 7002	*Synechococcus elongatus* PCC 7942	*Anabaena* sp. 7120
Genome	3.6 Mb Chromosome + 7 plasmids size ranging from 2.3 to 120 kb	3.0 Mb chromosome + 6 plasmids size ranging from 4.8 to 186 kb	2.7 Mb Chromosome + 46 kb plasmid	6.4 Mb chromosome + 6 plasmids size ranging from 5.6 to 408 kb
Description	Freshwater	Salt tolerant	Freshwater	Diazotrophic
Transformation	Naturally competent	Naturally competent	Naturally competent	Conjugation

**Table 2:** Chemical targets, promoters used, genes expressed, titer, and relevant central metabolites.

Chemical Target	Strain	Promoter(s) Used	Gene(s) Expressed	Gene Knockout(s)	Titer (mg/L)	Days of Cultivation	Relevant Central Metabolite	Comments	Ref.
Ethanol	PCC 6803	Prbc	*pdc*, *adh2*	*phb*	5500	26	Pyruvate	Two copies of *pdc*, optimized cultivation	[11]
Ethanol	PCC 6803	PpsbA2	*pdc*, *adh*		550	6	Pyruvate	Decarboxylation of pyruvate serves as efficient driving force	[12]
Ethanol	PCC 7942	Ptrc	*pduP*, *yqhD*		182	10	Acetyl-CoA	Oxygen tolerant aldehyde dehydrogenase	[13]
Ethanol	PCC 7942	PrbcLS	*pdc*, *adh*		0.02	7	pyruvate	expression of *pdc* and *adh* on plasmid	[14]
Isopropanol	PCC 7942	Ptrc	*thl-atoAD-adc-adh*		146	15	Acetyl-CoA	Medium optimization, 2 phase cultivation	[15]
Isopropanol	PCC 7942	Ptrc	*thl-atoAD-adc-adh*		26.5	9	Acetyl-CoA	decarboxylation of acetoacetate traps carbon to acetone	[16]
1-Butanol	PCC 7942	Ptrc/PLlacO1	*ter/nphT7*, *pduP*, *yqhD*, *crt*, *hbd*		317	12	Acetyl-CoA	Oxygen tolerant aldehyde dehydrogenase	[13]
1-Butanol	PCC 7942	Ptrc/PLlacO1	*ter/nphT7*, *bldh*, *yqhD*, *crt*, *hbd*		30	17	Acetyl-CoA	ATP driving force through acetoacetyl-CoA synthase	[17]
1-Butanol	PCC 7942	Ptrc/PLlacO1	*ter/atoB*, *adhE2*, *crt*, *hbd*		14.5	7	Acetyl-CoA	Dark anaerobic incubation	[18]
Isobutyraldehyde	PCC 7942	Ptrc/PlacO1	*kivd/alsS-ilvC-ilvD*		1100	8	Pyruvate	Decarboxylation of KIV serves as effective driving force. *In situ* product removal	[19]
Isobutanol	PCC 6803	Ptac	*kivD*, *adhA*		240	21	Pyruvate	Oleyol alcohol trap	[20]
Isobutanol	PCC 7942	Ptrc	*kvid*, *YqhD*		450	6	Pyruvate	Decarboxylation of KIV serves as effective driving force. *In situ* product removal	[19]
2-Methylbutanol	PCC 7942	Ptrc	*kivD*, *yqhD*, *cimA*, *leuBCD*		178	12	Pyruvate/acetyl-CoA	decarboxylation, native highly active AHAS	[21]
Fatty alcohol	PCC 6803	Prbc	*jojoba FAR*		0.2	18	Acetyl-CoA	using native fatty-acyl-ACP synthesis & expression of jojoba FAR	[22]
Fatty alcohol	PCC 6803	Prbc/PpsbA2	*jojoba FAR/aas*		0.17	10	Acetyl-CoA	expression of jojoba FAR & overexpression of acyl-ACP synthetase	[23]
1,2-Propanediol	PCC 7942	Ptrc	*sADH* (*C.beijerinkii*), *yqhD*, *mgsA*		150	10	Pyruvate	NADPH utilization	[24]
2,3-Butanediol	PCC 6803	Ptrc	*als*, *aldc*, *ar*		585	29	Pyruvate	Codon optimization	[25]
2,3-Butanediol	PCC 7942	PLlacO1	*alsS*, *alsD*, *adh*		2380	20	Pyruvate	pyruvate pool coupled to decarboxylation and product low toxicity	[26]
Glycerol	PCC 6803	Ptrc	*gpp2*		1068	17	DHAP	Salt stress can stimulate glycerol production even in wildtype for about 0.7 mM	[27]
Glycerol	PCC 7942	Ptrc	*gpp1*		1170	20	DHAP	aeration, thermodynamically favorable glycerol phosphatase	[28]
D-Lactate	PCC 6803	Ptrc	*gldA101*, *sth*		1140	24	Pyruvate	expression of transhydrogenase, codon optmized mutated glycerol dehydrogenase, addition of acetate helped production	[29]
D-Lactate	PCC 6803	Pcpc560	*Dldh*	*pta*,*phaCE*	1060	4	Pyruvate	knockout of PHB synthesis & acetate formation, expression codon optimized *ldh* from *Lactobacillus delbrueckii*	[30]
D-Lactate	PCC 7942	Ptrc	*ldhD*, *lldP*		829	10	Pyruvate	expression of lactate transporter, engineered Ldh to use NADPH	[31]
D-Lactate	PCC 7942	Plac	*ldhA*, *lldP*, *udhA*		55	4	Pyruvate	Expression of LldP protein from *E. coli* (transporter)	[32]
L-Lactate	PCC 6803	Ptrc	*ldh*		1800	40	Pyruvate	Long term production	[33]
L-Lactate	PCC 6803	Ptrc2/Ptrc2	*pk/ldh*	*knockdown PPC*	837	14	Pyruvate	codon optimization & natural copy	[34]
L-Lactate	PCC 6803	Ptrc/Ptrc	*ldh/sth*		288	14	Pyruvate	expression of transhydrogenase, *Bacillus subtilis* Ldh	[35]
L-Lactate	PCC 6803	PpsbA2	*ldh*, *ldhP*		15.3	18	Pyruvate	Tested various *ldh* genes.	[36]
3-Hydroxypropionate	PCC 7942	Ptrc	*mcr*, *msr*		659	16	Acetyl-CoA	selection of best performing malonate semialdehyde reductase (Msr), two NADPH utilizing steps	[37]
3-Hydroxypropionate	PCC 7942	Ptrc	*gpp1*, *dhaB*, *puuC*		31.7	10	DHAP	oxygen sensitive, Dark anaerobic with nutrient limitation	[28]
3-Hydroxybutyrate	PCC 6803	Ptca/Ptac	*tesB/phaA-phaB*		533	21	Acetyl-CoA	Nutrient limitation, NADPH	[38]
Itaconic acid	PCC 6803	Ptac	*cad*		14.5	16	Isocitrate	Expression of *cad*	[39]
p-Coumaric acid	PCC 6803	PpsbA2	*sam8*	*slr1573* (*laccase*)	82.6	4	Tyrosine	knockout of competing pathway for phenolic compound degradation	[40]
Fatty acids	PCC 6803	Ptrc/Pcpc/Prbc	*tesA*, *fatB1*, *fatB2/accBC/accDA*, *fat B2*	*aas*, *pta*, *phb genes*,(*see comments*)	197	2	Acetyl-CoA	construct six generation strain: extensive knock outs of PHB synthesis, peptidoglycan layer protein, hemolysin-like surface layer protein, cyanophycin synthesis	[41]
Fatty acids	PCC 7002	Ptrc/PpsbA1	*tesA/rbcLS*	*fadD*	131	20	Acetyl-CoA	Overexpression of rubisco	[42]
Fatty acids	PCC 7942	Ptrc	*tesA*	*aas*	45	20	Acetyl-CoA	knockout of acylACP synthetase blocks utilization of fatty acids	[43]
Fatty acids	PCC 7942	Ptrc/PpsbA1	*fat1/rbcLS*	*aas*	35	20	Acetyl-CoA	overexpression of ACCase hurts production	[44]
D-Mannitol	PCC 7002	PpsbA	*mtlD*, *mlp*	*glgA1*, *glgA2*	1100	12	F6P	codon optimization, artificial carbon sink	[45]
Hexose	PCC 7942	Ptrc	*glf*, *invA*, *galU*		45	5	Glc6P	expression of sugar transporter	[32]
Sucrose	PCC 7942	Ptrc	*cscB*	*invA*, *glgC*	2700	7	Glc6P	Salt stress, knockout of natural carbon/electron sink	[46]
Sucrose	PCC 6803	PpetE	*cscB*, *sps*, *spp*, *ugp*	*ggpS*, *ggtCD*	140	10	Glc6P	Salt stress, knockout of competing pathways, expression of sucrose synthesis genes	[47]
Glucosylglycerol	PCC 6803	--	--	*ggtCD*, *ggpR*	981	24	G3P/Glc6P	salt shock, hypoosmotic shock	[48]
Ethylene	PCC 6803	Ptrc	*efe*		240 nL/mL/d		α-Ketoglutarate	compared various promoters, used plasmid based expression	[49]
Ethylene	PCC 6803	PpsbA	*efe*		171 mg/L/d		α-Ketoglutarate	Multiple copies of EFE	[50]
Ethylene	PCC 7942	Ptrc	*ACS-Ctdoc-ACO-Acdoc-Cip2*		81.6 nL/mL/d/OD		SAM	Chimeric protein fusion	[51]
Ethylene	PCC 7942	PpsbA1	*efe*		10.82 μL/mL/D/OD		α-Ketoglutarate	choose a strong promoter site in 7942 and rpsl2-mediated gene replacement	[52]
Isoprene	PCC 6803	PpsbA2	*IspS* (*Pueraria montana*)		0.35	8	G3P/Pyruvate	Gaseous/aqueous two-phase photobioreactor	[53]
Isoprene	PCC 6803	PpsbA2	*IspS*, *hmgS*, *hmgR*, *fni*, *mk*, *pmd*, *pmk*		0.3	8	Acetyl-CoA	expression of both pathways to IPP increases isoprene production	[54]
Isoprene	PCC 6803	PpsbA2	*IspS* (*Pueraria montana*)		50 μg/gDCW/d		G3P/Pyruvate	Expression of isoprene synthase	[55]
Limonene	PCC 6803	Ptrc	*limS* (*Schizonepeta tenuifolia*), *dxs*, *crtE*, *ipi*		1	30	G3P/Pyruvate	codon optimization, enhancing flux through MEP pathway	[56]
Limonene	PCC 7002	PcpcBA	*limS* (*Mentha spicata*)		4	4	G3P/Pyruvate	product trap by dodecane overlayLan	[57]
Limonene	PCC 7120	Pnir::PpsbA1	*limS* (*Picea sitchensis*), dxs, ipphp, gpps		0.52	12	G3P/Pyruvate	enhancing flux through MEP pathway by gene overexpression, high light density	[58]
Farnesene	PCC 7120	Pnir, PpsbA1	*faS* (*Picea abies*)		0.31	15	G3P/Pyruvate	codon optimization	[59]
Bisabolene	PCC 7002	PcpcBA	*bis* (*Abies grandis*)		0.6	4	G3P/Pyruvate	product trap by dodecane overlay	[57]
Tocopherols	PCC 6803	PnirA	*hpd* (*Arabidopsis thaliana*)		0.250 mg/gDCW	12	G3P/Pyruvate	Nitrate inducible promoter	[60]
β-Caryophyllene	PCC 6803	PpsbA2	*QHS1*		0.046	7	G3P/Pyruvate	use similar pathway in 6803 to produce plant's second metabolite, only need few key enzyme	[61]
β-Phellandrene	PCC 6803	PpsbA2-trc-T7	*PLHS* (*Lavandula angustifolia*)		0.9	2	G3P/Pyruvate	codon optimization, High light with psba2-trc-T7 fused promoter	[62]
β-Phellandrene	PCC 6803	PpsbA2	*PHLS* (*Lavandula angustifolia*)		0.2	8	G3P/Pyruvate	codon optimization	[63]
Dihydroxyacetone	PCC 7942	Ptrc	*gpp1*, *dhaD*		78.6	16	DHAP	NAD-dependent DhaD could not efficiently reduce glycerol	[28]
Acetone	PCC 6803	Prbc/Pcpc	*cftAB/adc*	phaCE,pta	36	4	Acetyl-CoA	Increasing acetyl-CoA pool	[64]
Alkanes	PCC 7120	Pado	*aar*, *ado* (*A. halophytica*)		1.25 mg/gDCW	5	Acetyl-CoA	Salt stress	[65]

## 2. Short Chain Alcohols

### 2.1. Ethanol

**Figure 2:**
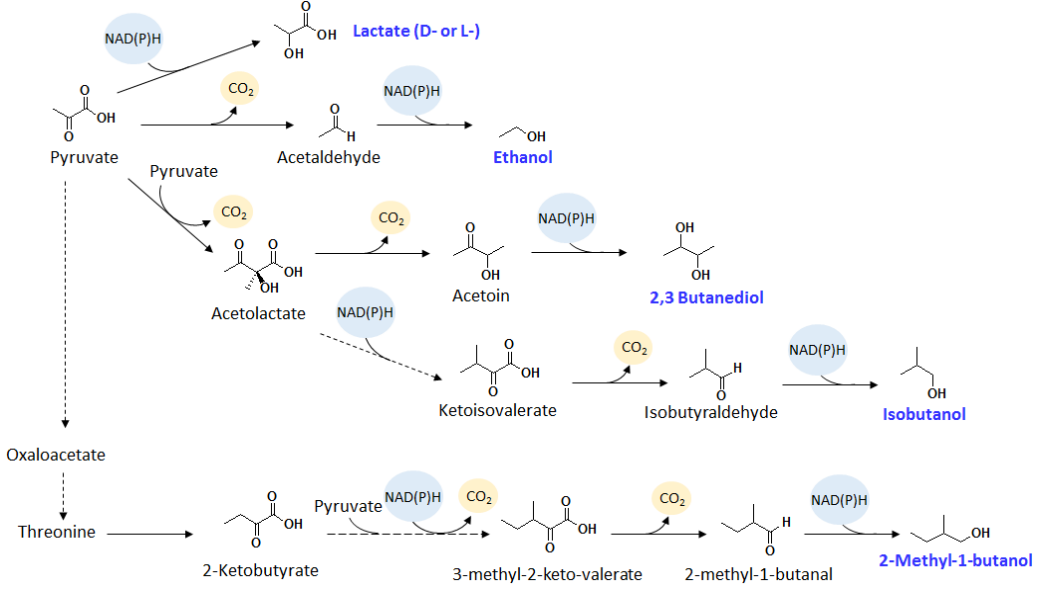
Schematics of pyruvate derived bioproducts. Dashed lines represent multiple enzymatic steps. Evolution of CO_2_ serves as effective driving force for product formation.

Some cyanobacteria naturally produce small amounts of ethanol under fermentative conditions [66]. However, genetic engineering was necessary to increase the productivity and enable production under photosynthetic conditions. Ethanol production from PCC 7942 [14] has been demonstrated as early as the late 20th century. While the productivity (54 nmol/L/d/OD_730_) is relatively low, it is the first alcohol produced from genetically modified cyanobacteria. Ten years later, an ethanol production titer of 550 mg/L with a productivity of 5.2 mmol/L/d/OD_730_ from PCC 6803 [12] was achieved using photobioreactor. In both PCC 7942 and PCC 6803, ethanol was produced using the pyruvate decarboxylation pathway from *Zymomonas mobilis* (Figure 2), employing pyruvate decarboxylase (Pdc) and alcohol dehydrogenase (Adh). Pyruvate decarboxylation is an irreversible reaction and therefore provides a strong driving force for acetaldehyde formation. Decarboxylation is a good example of a powerful driving force for product formation because the gaseous CO_2_ leaves the system, pulling the carbon flux towards the product. It is uncertain why the first ethanol report using PCC 7942 was significantly less successful than using PCC 6803. However, it is possible that the gene expression was more stable in the PCC 6803 study as the two genes were introduced into the genome rather than using a plasmid based system. 

To further increase the titer of ethanol production, additional optimization was carried out [11]. In this study, PCC 6803 endogenous alcohol dehydrogenase was expressed instead of that from *Z. mobilis*. In addition, the expression of multiple copies of the *pdc* and *adh* increased the gene dosage. *In situ* ethanol removal using gas stripping further improved ethanol productivity. As ethanol is continuously removed from the culture, equilibrium further favors to push carbon flux towards ethanol. Through both genetic and cultivation optimizations, ethanol production reached a titer of 5.5 g/L in 26 days, representing the highest biochemical production titer demonstrated in the cyanobacterial engineering literature.

**Figure 3:**
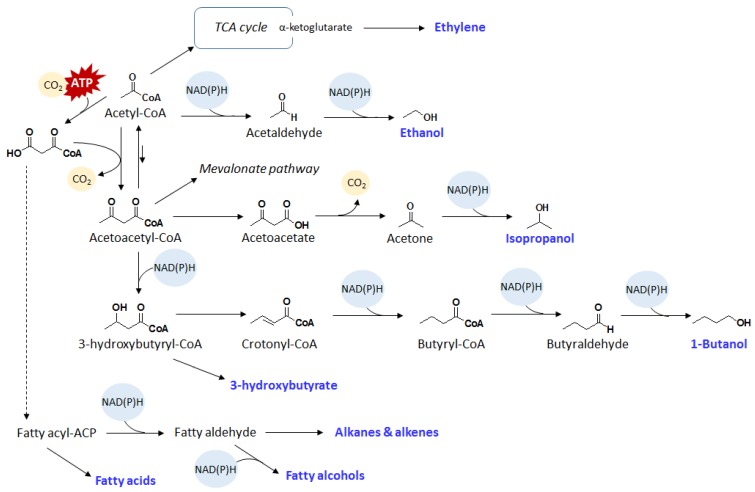
Schematics of acetyl-CoA derived bioproducts. Dashed lines represent multiple enzymatic steps.

Alternatively, ethanol can also be produced from acetyl-CoA (Figure 3). Ethanol production from acetyl-CoA is mediated by a bifunctional aldehyde/alcohol dehydrogenase AdhE in a reversible reaction. AdhE is known to be oxygen sensitive, and therefore its functional expression in cyanobacteria would be difficult. To bypass AdhE, the expression of separate oxygen tolerant CoA-acylating aldehyde dehydrogenase and alcohol dehydrogenase effectively converts acetyl-CoA to ethanol via acetaldehyde under aerobic conditions [13]. By expressing PduP, an oxygen tolerant CoA-acylating aldehyde dehydrogenase from the 1,2-propanediol degradation pathway from *Salmonella enterica*, and YqhD, a NADPH dependent alcohol dehydrogenase, PCC 7942 was engineered to produce ethanol from acetyl-CoA. The resulting strain produced 182 mg/L of ethanol in 10 days. It is worth noting that this pathway requires two reducing cofactors. While the consumption of two reducing cofactors drives the synthesis of ethanol, these results potentially indicate that decarboxylation serves as a stronger driving force for ethanol production. 

### 2.2. Isopropanol

Isopropanol is a natural fermentation product from certain microbes. Industrially, isopropanol is widely used as a solvent and can be dehydrated into propylene, a petroleum-derived monomer for the production of the widely used plastic, polypropylene. The production pathway for isopropanol begins with acetyl-CoA condensation to acetoacetyl-CoA, which is followed by the removal of the CoA moiety to form acetoacetate. Acetoacetate is then decarboxylated into acetone, which is subsequently reduced to isopropanol (Figure 3). Isopropanol production pathway and genes were introduced into PCC 7942 [16]. Despite the expectation that the isopropanol pathway has a decarboxylation as driving force, the resulting strain was not able to produce isopropanol under photosynthetic conditions. Further analysis through acetate feeding showed that the acetyl-CoA level in PCC 7942 is insufficient to support isopropanol production. As an alternative approach, cyanobacteria expressing isopropanol production genes were incubated in the dark without nitrogen and phosphorus. Under nutrient limiting conditions, the acetyl-CoA flux was hypothesized to increase. As a result, 26.5 mg/L of isopropanol was observed [16]. With further optimization of cultivation conditions, isopropanol production has been increased to 146 mg/L [15].

In another study aimed at producing acetone, the precursor to isopropanol, using engineered PCC 6803, acetate forming genes were disrupted to increase the pool of intracellular acetyl-CoA [64]. This approach increased the acetone production six-fold reaching a titer of 36 mg/L in four days. Together, these results indicated that in addition to decarboxylation, increasing the substrate pool is also an important design principle.

### 2.3. Isobutanol

The isobutanol production pathway utilizes valine biosynthesis. The valine precursor, ketoisovalerate, is decarboxylated to isobutyraldehyde, which can be directly reduced into isobutanol via alcohol dehydrogenase (Figure 2). An isobutanol pathway without alcohol dehydrogenase was first expressed in PCC 7942 [19]. The resulting strain produced isobutyraldehyde with a titer of 1.1 g/L using *in situ* product removal through gas stripping. Aldehydes are generally toxic to life. Therefore, *in situ* product removal lowers the toxic effect of isobutyraldehyde production to cyanobacteria. Isobutyraldehyde was further converted to isobutanol in PCC 7942 with additional expression of alcohol dehydrogenase YqhD. The resulting strain produced 450 mg/L of isobutanol in six days.

In another study, the isobutanol pathway was expressed in PCC 6803 [20]. In shake flask experiment, 90 mg/L of isobutanol was observed in six days. Further improvement using oleyl alcohol as a product trap to remove butanol from the culture increased the production titer up to 240 mg/L. This result also demonstrated the usefulness of product removal.

### 2.4. 1-Butanol

1-Butanol is a natural fermentation product from certain *Clostridium* species. 1-Butanol production in non-native organisms has been difficult due the enzyme oxygen sensitivity and less well-defined redox reactions. One of the key enzymes of the *Clostridium* butanol pathway is the butyryl-CoA dehydrogenase electron transferring protein complex. This enzyme was difficult to express in heterologous organisms because it requires association with ferredoxin and is oxygen sensitive. This difficulty was overcome by replacing this enzyme complex with a trans-enoyl-CoA reductase (Ter) [67,68]. Ter directly utilizes NADH as the reducing cofactor for the reduction of crotonyl-CoA to butyryl-CoA (Figure 3) with a large negative change in free energy, making it irreversible and thus serving as an effective driving force for this pathway.

This modified 1-butanol pathway was subsequently transferred to PCC 7942 [18]. However, the resulting strain did not produce 1-butanol under photosynthetic conditions. 1-Butanol was produced only under dark anaerobic conditions [18]. One difficulty was that that the first reaction of the pathway catalyzed by thiolase is thermodynamically unfavorable with K_eq_ of 10^−5^. To circumvent this difficult step, an alternative route using malonyl-CoA was constructed [17]. Malonyl-CoA reacts with acetyl-CoA to form acetoacetyl-CoA in an irreversible reaction catalyzed by acetoacetyl-CoA synthase. Naturally, malonyl-CoA is the ATP activated product of acetyl-CoA through the acetyl-CoA carboxylase. Combining the reactions catalyzed by acetyl-CoA carboxylase and acetoacetyl-CoA synthase, the net reaction is two acetyl-CoA condensation to acetoacetyl-CoA at the expense of one ATP. This ATP hydrolysis serves as an energy input to convert the thermodynamically unfavorable acetyl-CoA condensation into a favorable reaction. The expression of acetoacetyl-CoA synthase with the butanol production pathway enabled the direct photosynthetic production of 1-butanol with a titer of 30 mg/L.

1-Butanol production was further improved by the expression of an oxygen tolerant CoA-acylating aldehyde dehydrogenase [13]. The *Clostridium* aldehyde dehydrogenase is oxygen sensitive, which prohibits its functional expression in cyanobacteria under photosynthetic conditions. To solve this difficulty, the oxygen tolerant CoA-acylating aldehyde dehydrogenase of the 1,2-propanediol degradation pathway from *S. enterica* was used to replace the *Clostridium* aldehyde dehydrogenase. The resulting strain was able to produce about 300 mg/L of 1-butanol in 12 days, representing a 10-fold increase compared to the 1-butanol produced using the *Clostridium* aldehyde dehydrogenase. This result shows that the oxygen tolerance of enzymes is also very important for biochemical production using cyanobacteria.

### 2.5. 2-Methyl-1-Butanol

In addition to isobutanol, other branched short chain alcohols are also biofuel candidates, which have superior properties over ethanol. 2-Methyl-1-butanol (2MB) is derived from 2-keto-3-methylvalerate (KMV), the precursor to isoleucine, through the same keto acid type pathway as the isobutanol production (Figure 2). KMV is derived from 2-ketobutyrate through the isoleucine biosynthesis. 2-Ketobutyrate is derived from either the cyanobacterial native threonine pathway or heterologous citramalate pathway. In a study where the citramalate pathway was expressed in PCC 7942 together with a ketoacid decarboxylase and an alcohol dehydrogenase, 2MB was produced with a titer of 178 mg/L in 12 days [21]. Interestingly, the isoleucine biosynthesis was not overexpressed, and yet 2MB was produced instead of 1-propanol. This result was surprising as the expression of the same set of genes in *E. coli* resulted in mostly 1-propanol production [69]. This study indicated that the native isoleucine genes were highly expressed and active in PCC 7942. The 2MB production pathway is analogous to the isobutanol pathway, therefore exhibiting similar characteristics and driving force properties. However, the production titer was lower than that of isobutanol. This is potentially due to lower substrate (2-ketobutyrate) pool and higher product toxicity.

## 3. Fatty Acids and Hydrocarbons

### 3.1. Fatty Acids

Biodiesel is typically derived from chemically reacting lipids with short chain alcohols such as methanol, ethanol or propanol, into corresponding fatty esters. Instead of producing triacylglycerides, microbial systems can be engineered to directly produce fatty acids, which can also be esterified. Fatty acid production from PCC 6803 has been demonstrated with a titer of 197 mg/L in two days [41]. The expression of TesA, *E. coli* thioesterase with N-terminus periplasmic directing sequence deleted, and codon optimized plant thioesterases releases fatty acids from their fatty acyl-(acyl-carrier-protein) (ACP) precursors. Together with the deletion of genes encoding for fatty acid activation, polyhydroxybutyrate (PHB), S-layer, cyanophycin, and acetate biosynthesis, the final titer of fatty acid produced was increased from 1.8 mg/L to 197 mg/L. Liu *et al.* [70] further demonstrated a strategy which enables cyanobacteria to degrade cellular membranes and release free fatty acids upon CO_2_ limitation. This fatty acid releasing strategy was accomplished by the expression of lipases under the promoter control of native inorganic carbon uptake system. 

In another study, PCC 7002 was engineered to produce fatty acids with a titer of 131 mg/L in 20 days [42]. In particular, it was noted that PCC 7002 has a higher tolerance to fatty acids. In this study, it was found that the expression of heterologous RuBisCo, the primary enzyme responsible for CO_2_ fixation in CBB pathway, was able to increase the target productivity. Similar effect was also observed by Atsumi *et al.* [19] in the isobutanol production. While successful in increasing biochemical productivity, the effect of RuBisCo overexpression in carbon fixation rate has yet to be clarified.

### 3.2. Fatty Alcohols and Hydrocarbons

Many species of cyanobacteria naturally produce small quantities of hydrocarbons [71]. The hydrocarbon production pathway and its enzymes were identified [72,73]. Fatty acyl-ACP, the end product of fatty acid elongation, is reduced to fatty aldehyde by fatty acyl-ACP reductase. Fatty aldehyde decarbonylase then catalyzes the conversion of fatty aldehydes into alkanes or alkenes with formate as a byproduct. 

Engineered PCC6803 has been demonstrated to produce fatty alcohols with a titer of around 0.2 mg/L through the expression of fatty acyl-CoA reductase from jojoba [22]. While hydrocarbon biosynthesis is natural to many cyanobacteria, overexpression of the genes involved in alkane biosynthesis in cyanobacteria has been used to increase alkane productivity [65,74,75]. However, compared to the higher productivity of fatty acids, both fatty alcohol and hydrocarbon productions are relatively low, indicating the presence of bottlenecks. It is likely that the key enzyme fatty acyl-ACP reductase is limiting, as the flux to fatty acyl-ACP is capable of supporting higher flux production of fatty acids. As an alternative to using acyl-ACP reductase, liberation of fatty acids followed by activation to acyl-CoAs allows the use of CoA-acylating aldehyde dehydrogenase, which has been shown to reduce to fatty aldehyde more efficiently in *E. coli* [76]. However, this pathway has yet to be tested in cyanobacteria.

## 4. Olefins

### 4.1. Ethylene

Microbial ethylene production is catalyzed by the ethylene forming enzyme (Efe). Efe utilizes α-ketoglutarate and produces succinate as a byproduct. Metabolic engineering of cyanobacteria for the production of ethylene has been reported as early as the 1990s [77,78]. However, the productivities reported were relatively low due to the instability of *efe* within the cyanobacteria as *efe* becomes truncated through successive subculturing [52]. This problem was addressed by eliminating the potential “hot spots” for mutation within *efe* through codon optimization [50]. In addition, *efe* was expressed in multiple copies, increasing gene dosage and therefore the overall enzymatic activity. The resulting engineered PCC 6803 strain was able to achieve significant improvement of ethylene production with a relatively high peak productivity of 171 mg/L/d. Ethylene production is the one of the few example which achieves higher productivity utilizing acetyl-CoA. PCC 6803 has also been engineered to co-utilize xylose, an abundant bioresource, for aiding ethylene production [79]. In addition, cyanobacterial ethylene production has also been achieved through the expression of plant ethylene formation pathway that utilizes S-adenyl-methionine (SAM) in two reaction steps [51]. SAM is first converted to methylthioadenosine and 1-aminocyclopropane-1-carboxylate. 1-aminocyclopropane-1-carboxylate is subsequently converted to ethylene, producing hydrogen cyanide and CO_2_ as byproducts. The resulting strain achieved 81.6 nL/mL/d/OD [51]. 

### 4.2. Isoprene

**Figure 4:**
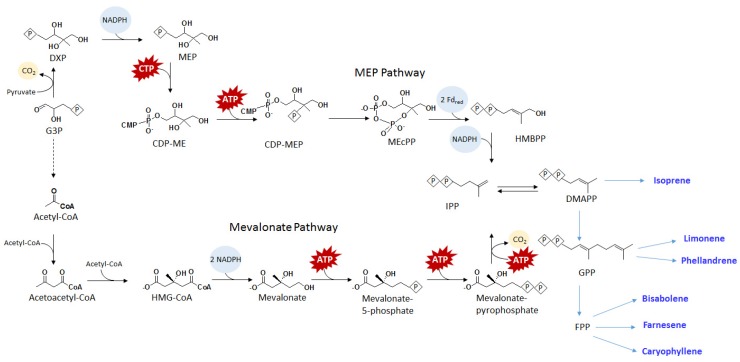
Schematics of mevalonate and MEP pathways for synthesizing isoprenoids and terpenoids. Symbol P inside a diamond represents phosphate. For abbreviations, see below in the abbreviations section.

Cyanobacteria and most other bacteria use the methyl-erythritol-4-phosphate (MEP) pathway (Figure 4) for the biosynthesis of isoprenoids. Isoprene, a five carbon diene, is the component of polyisoprene or latex, which is also an important target for renewable chemical production. Isoprene is synthesized from Dimethylallyl pyrophosphate (DMAPP) using isoprene synthase. Isoprene synthase is a natural enzyme found in plants, particularly in poplar and kudzu vine. Through heterologous expression of isoprene synthase in PCC 6803 [55], direct photosynthetic isoprene production was achieved. However, the productivity achieved (50 µg/gDCW/d) was relatively low. To increase the productivity of isoprene production, the mevalonate pathway (Figure 4) was co-expressed with isoprene synthase in PCC 6803 [54]. With the additional knockout of glycogen synthesis, the resulting strain was able to produce up to 300 µg/L of isoprene in eight days, representing several fold improvement over the strain expressing isoprene synthase only. 

### 4.3. Terpenoids

Terpenoids represent a large class of bioactive molecules. In particular, some terpenoids are excellent targets for biofuel. Thus far, photosynthetic production of monoterpenes and sesquiterpenes has been demonstrated using engineered cyanobacteria. The chemical targets and their corresponding production titers are listed in Table 2. Monoterpene production follows the same MEP pathway as isoprenoids (Figure 4). DMAPP and its isomer isopentenyl diphosphate (IPP) react to form the ten carbon metabolite geranyl diphosphate (GPP). Both limonene and β-phellandrene are produced directly from GPP using limonene synthase and β-phellandrene synthase, respectively. Cyanobacterial engineering strategies for limonene and β-phellandrene production include the expression of MEP pathway genes for increasing pathway flux [58], dodecane overlay as product trap [57], and increased expression of limonene synthase through promoter engineering [62]. However, the resulting titers of monoterpene productions remain relatively low (around 1 mg/L). Sesquiterpene biosynthesis elongates GPP into farnesyl diphosphate (FPP). Similar to the production of monoterpenes, biosynthesis of sesquiterpenes such as β-caryophyllene, farnesene, and bisabolene requires only the corresponding synthases. Recently, diterpene manoyl oxide, precursor to forskolin, production has been reported using engineered PCC 6803 [80]. Resulting titers (Table 2) of sesquiterpenes produced using engineered cyanobacteria were relatively low compared to other bioproducts. The bottleneck of terpenoids production using cyanobacteria has yet to be determined. 

## 5. Organic Acids

### 5.1. Lactate

Lactate fermentation is widely practiced both in food preparation and for renewable plastics. Among the current bioproducts produced by engineered cyanobacteria, lactate is one of the most extensively studied biochemical productions in cyanobacteria and produced with high productivity. Both isomers of lactate have been produced using cyanobacteria. Some engineering strategies involved in lactate production include the expressions of lactate transporters [32], transhydrogenase for converting NADPH produced by photosynthesis into NADH [35], and engineered lactate dehydrogenase (Ldh) for direct NADPH utilization [31], overexpression of pyruvate kinase to trap carbon flux into pyruvate from PEP [34], and knockouts of competing carbon sinks [30]. Currently, the highest lactate production demonstrated is 1.8 g/L in 40 days. In addition, waste water from anaerobic digest rich in organics, nitrogen, and phosphorus, was used to aid photosynthetic lactate production [81] in PCC 6803, illustrating the beneficial application of mixotrophic cultivation. 

### 5.2. 3-Hydroxybutyrate

3-Hydroxyacids are the monomers of polyhydroxyalkanoates, which are biocompatiable and biodegradable thermoplastics or elastomeric materials. 3-Hydroxybutyrate (3HB) production pathway follows the butanol and isopropanol biosynthesis (Figure 3). Acetyl-CoA is used as the cellular metabolite to form acetoacetyl-CoA, which is followed by reduction to 3-hydroxybutyryl-CoA. Subsequently, 3-hydroxybutyryl-CoA undergoes a thioesterase catalyzed reaction to liberate 3HB and CoA. 3HB production pathway genes were introduced into PCC 6803 [38]. Similar to the isopropanol and butanol reports, production of 3HB is limited under photosynthetic conditions. Nevertheless, as nutrients become limited through consumption, the intracellular acetyl-CoA pool increases. The acetyl-CoA pool naturally increases in PCC 6803 upon nutrient depravation in order to synthesize PHB, a natural carbon storage. As increasing acetyl-CoA pool drives 3HB production, the resulting strain produced up to 533 mg/L of 3HB in 21 days. 

### 5.3. 3-Hydroxpropionate 

**Figure 5:**
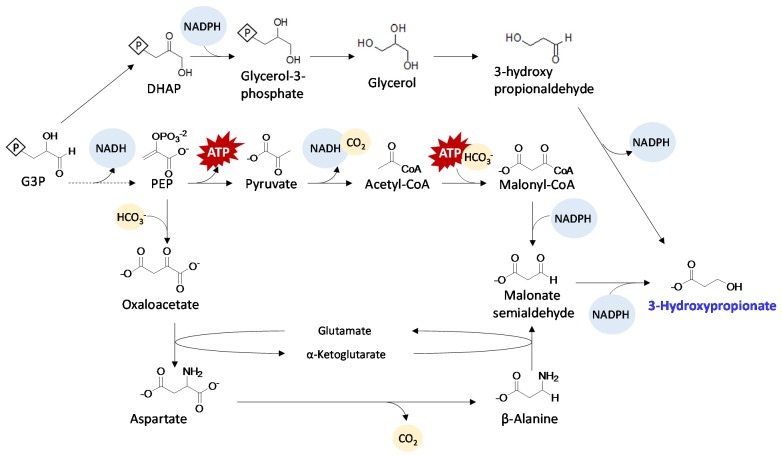
Schematics of 3HP production pathways.

One of the most well characterized 3-hydroxypropionate (3HP) production pathway utilizes glycerol as its precursor (Figure 5). Glycerol dehydrates to 3-hydroxypropionylaldehyde, which is subsequently oxidized to 3HP. PCC 7942 was engineered to produce 3HP with a titer of 31.7 mg/L in 10 days [28]. In this work, the glycerol dehydratase used was oxygen sensitive. As a result, 3HP was only produced upon incubation in dark anaerobic conditions. The oxygen sensitivity of glycerol dehydratase would have to be addressed before the direct photosynthetic 3HP production can be realized using this pathway.

Using a different pathway, PCC 7942 was engineered to synthesize 3HP via two step reduction (Figure 5) from malonyl-CoA [37]. This pathway utilizes genes from the 3-hydroxypropionate/4-hydroxybutyrate carbon fixation pathway. The resulting strain produced 665 mg/L of 3HP, 10-fold higher than that produced by strains expressing the glycerol dependent pathway. In the same study, a synthetic pathway was constructed to produce 3HP via β-alanine (Figure 5). The resulting strain expressing this β-alanine dependent pathway produced 186 mg/L of 3HP. Compared to the glycerol dependent pathway for 3HP production, these two pathways do not have any known oxygen sensitivity. Therefore, these results again demonstrated the importance of using oxygen tolerant enzymes for cyanobacterial engineering. It is worth noting that 3HP is toxic at low concentrations to cyanobacteria [82]. Further strain engineering would be necessary for increasing 3HP productivity.

### 5.4. p-Coumaric Acid 

Phenylpropanoids are a large group of secondary metabolites produced by plants with some having anti-inflammatory and antiviral properties. p-Coumaric acid is the precursor to these phenylpropanoids and can be produced via one step deamination from tyrosine. Recently, PCC 6803 was engineered to express tyrosine ammonia lyase (Tal) [40]. Interestingly, expression of Tal alone did not allow production of p-coumaric acid. Further analysis revealed the presence of a native laccase in PCC 6803 that degrades p-coumaric acid. Upon knocking out the laccase gene, the resulting strain was able to produce 82.6 mg/L of p-coumaric acid. The Tal catalyzed reaction serves as a driving force for production of p-coumaric acid as the liberation of NH_3_ is thermodynamically favorable. A similar driving force has also been employed in the development of protein based biofuel production [83]. PCC 6803 was further engineered to convert p-coumaric acid into caffeic acid [84], also an important chemical with many bioactive properties and a precursor to many other bioactive products.

## 6. Sugars

Cyanobacteria are generally less tolerant to chemicals than other heterotrophic hosts such as *E. coli* and yeast, hindering its industrialization. Therefore, as an alternative approach, cyanobacteria are engineered to produce soluble sugars that are not toxic to cyanobacteria and can be directly utilized by heterotrophs. Sucrose is a natural product produced by cyanobacteria as an osmoprotectant. Sucrose can accumulate intracellularly at a concentration of around 300 mM [85,86], representing a large amount of potential sugar that can be produced. To facilitate sucrose transport across the cellular membrane, sucrose permease (CscB), a sucrose/proton symporter, was expressed in PCC 7942 [46]. As cyanobacteria generate proton gradient from inside of thylakoid membranes and alkalinize the environment, it facilitates removal of sucrose from cytoplasm. The expression of CscB and knockout of glycogen synthesis enabled the production of sucrose up to 2.7 g/L in engineered PCC 7942, representing a significant portion of carbon portioned into sucrose production. Glucose and fructose have also been produced by PCC 7942 [32] upon expression of invertase, which hydrolyzes sucrose. 

In another study, sucrose production through metabolic engineering of PCC 6803 has been examined [47]. It was found that the sucrose accumulation of PCC 6803 was not as high as PCC 7942. Through overexpressing sucrose synthesis genes and knocking out the genes for glucosylglycerol formation, the resulting strain produced 140 mg/L of sucrose in 10 days.

Mannitol is a sugar alcohol used in the pharmaceutical and food industries. To produce mannitol using recombinant cyanobacteria, PCC 7002 was engineered to express mannitol-1-phosphate dehydrogenase (MtlD) and mannitol-1-phosphatase (Mlp) [45]. Together with glycogen synthesis knocked out, the resulting strain produced 1.1 g/L of mannitol. Interestingly, it was noted that mannitol was exported to the culture medium through an unknown mechanism. These relatively high flux photosynthetic sugar/sugar alcohol production studies indicated that the flux for sugar synthesis is naturally high in cyanobacteria as the overexpression of the genes related to the synthesis of upper glycolytic intermediates were not necessary. 

## 7. Diols and Polyol

### 7.1. 2,3-Butanediol

2,3-Butanediol is a natural fermentative product and synthesized from pyruvate. Using the same acetolactate synthase from the isobutanol biosynthesis, the production of 2,3-butanediol was achieved also with relatively high flux in both PCC 7942 [26] and PCC 6803 [25]. The engineered 2,3-butanediol producing strain from PCC 7942 was able to produce over 2.4 g/L of 2,3-butanediol in 20 days, representing the second highest biochemical production titer reported. Subsequent improvements were made to balance the expression of pathway genes via combinatorial optimization of 5′UTR [87] and increase pyruvate pool by overexpressing enzymes between 3-phosphoglycerate to pyruvate [88]. Interestingly, one study showed that there is a hidden constitutive promoter within both *alsS* and *adh* [89]. This observation demonstrated that results obtained from traditional model organisms such as *E. coli* may or may not always translate into cyanobacteria because of both genetic and metabolic differences.

### 7.2. 1,2-Propanediol

Similar to other diols, 1,2-propanediol is an important chemical feedstock. The pathway to 1,2-propanediol biosynthesis starts with DHAP, which is converted to methylglyoxal via methylglyoxal synthase. Methylglyoxal is then converted to 1,2-propanediol in two steps using two alcohol dehydrogenases. NADPH utilizing alcohol dehydrogenases were cloned into PCC 7942 together with methylglyoxal synthase. The resulting strain produced 1,2-propanediol with a titer of 150 mg/L in 10 days [24]. 

### 7.3. Glycerol

Glycerol has been extensively studied as a carbon source for producing many C3 chemicals such as 1,3-propanediol and 3-hydroxypropionate [90]. Glycerol is naturally synthesized in yeast as an osmoprotectant. Its production pathway starts with DHAP, which is then reduced to glycerol-3-phosphate. Once hydrolyzed, glycerol-3-phosphate becomes glycerol (Figure 5). The expression of *Saccharomyces cerevisiae* phosphoglycerol phosphatase (Gpp) in both PCC 6803 [27] and PCC 7942 [48] have yielded direct photosynthetic production of glycerol. In the PCC 6803 study, increasing the medium salt concentration and the expression of Gpp enabled production of glycerol up to 1.06 g/L. It was noticed that salt stress alone was able to induce glycerol synthesis in the wild type [27]. In the PCC 7942 study, glycerol production reached a titer of 1.17 g/L. In this study, glycerol further served as a precursor for producing 3-hydroxypropionate and dihydroxyacetone using engineered PCC 7942. Co-cultivation of glycerol producing cyanobacteria with *Klebsiella pneumoniae* also enabled the production of 1,3-propanediol. Glycerol productivity achieved in both studies are relatively high compared to other bioproducts. In addition to the non-toxic effect of glycerol to cyanobacteria, naturally high flux to glycerol-3-phosphate, especially under salt stress, contributed to glycerol’s high flux biosynthesis. Furthermore, the dephosphorylation is also an irreversible reaction *in vivo*, further providing the driving force necessary for glycerol production.

## 8. Conclusions

Photoautotrophic production of biochemicals using metabolic engineered cyanobacteria is an attractive direction towards sustainability. Analysis of the chemical productions with higher titers often indicated that an effective metabolic driving force is necessary. It is worth noting that biochemical productions with titers exceeding 1 g/L originate from pyruvate and other metabolites of sugar metabolism, potentially indicating that these metabolites are more accessible to heterologous pathways than acetyl-CoA. While some production pathways naturally have driving forces, many others do not. In those cases, a driving force may be engineered to increase the favorability of the pathway. Improving on these design strategies, the performance of cyanobacterial biochemical factories may also be improved.

## Abbreviations

G3P: glyceraldehyde-3-phosphate
DXP: 1-Deoxy-D-xylulose 5-phosphate
MEP: methylerythritol 4-phosphate
CDP-ME: 4-diphosphocytidyl-2-C-methylerythritol
CDP-MEP: 4-diphosphocytidyl-2-C-methyl-D-erythritol 2-phosphate
MEcPP: 2-C-methyl-D-erythritol 2,4-cyclopyrophosphate
HMBPP: (E)-4-Hydroxy-3-methyl-but-2-enyl pyrophosphate
IPP: Isopentenyl pyrophosphate
DMAPP: Dimethylallyl pyrophosphate
GPP: Geranyl pyrophosphate
FPP: Farnesyl pyrophosphate
HMG-CoA: 3-hydroxy-3-methylglutaryl-CoA
F6P: fructose-6-phosphate
Glc6P: glucose-6-phosphate
DHAP: dihydroxyacetone phosphate
SAM: S-adenosylmethionine
